# *Achyranthis radix* Extract-Loaded Eye Drop Formulation Development and Novel Evaluation Method for Dry Eye Treatment

**DOI:** 10.3390/pharmaceutics12020165

**Published:** 2020-02-17

**Authors:** Sung-Jin Kim, Bongkyun Park, Hyun Wook Huh, Young-Guk Na, Minki Kim, Mingu Han, Hyunmin Lee, Thi Mai Anh Pham, Hong-Ki Lee, Jae-Young Lee, Chan-Sik Kim, Jong-Suep Baek, Cheong-Weon Cho

**Affiliations:** 1 College of Pharmacy and Institute of Drug Research & Development, Chungnam National University, 99 Daehak-ro, Yuseong-gu, Daejeon 34134, Korea; lanop@naver.com (S.-J.K.); hhw3573@nate.com (H.W.H.); youngguk@cnu.ac.kr (Y.-G.N.); zkzkang@naver.com (M.K.); linuxfalcon@naver.com (M.H.); gusals2218@naver.com (H.L.); phammaianhdkh68@gmail.com (T.M.A.P.); dvmlhk@gmail.com (H.-K.L.); jaeyoung@cnu.ac.kr (J.-Y.L.); 2 Clinical Medicine Division, Korea Institute of Oriental Medicine, Daejeon 34054, Korea; bkpark@kiom.re.kr (B.P.); chskim@kiom.re.kr (C.-S.K.); 3 Korean Convergence Medicine, University of Science and Technology (UST), Daejeon 34054, Korea; 4 Department of Herbal Medicine Resource, Kangwon National University, 346 Hwangjo-gil, Dogye-eup, Samcheok-si, Gangwon-do 25949, Korea; jsbaek@ntu.edu.sg

**Keywords:** *Achyranthis radix* extract, eye drop, lubricants, pigmentation, conjunctival epithelial cell

## Abstract

Recently, *Achyranthis radix* extract has been studied as a therapeutic agent for dry eye disease that occurs from fine dust. The aim of this study was the development of *Achyranthis radix* extract-loaded eye drop formulations using lubricants, generally used for artificial tear eye drops. Ecdysterone was used as a marker compound for *Achyranthis radix* extract and 1% *Achyranthis radix* extract solution contained 14.37 ± 0.04 μg/mL of ecdysterone. Before formulation studies, a new method was performed to evaluate pigmentation, which might be caused by eye drops of herbal extract. A comparative study of the water retention ability of each formulation and ability to prevent the death of conjunctival epithelial cells in dry conditions was conducted. Moreover, treatment of *Achyranthis radix* extract (USL) eye drop formulation exhibited a significant inhibitory effect on inflammation in a concentration-dependent manner. The long-term and accelerated stability tests showed that lubricants could contribute to the stability of herbal extracts in solution. In conclusion, hyaluronic acid showed a good effect on the development of eye drop formulation using *Achyranthis radix* extracts for treating dry eye disease.

## 1. Introduction

Dry eye disease (DED) is the condition of having dry eyes, in which the eye surface is damaged due to lack of tears, excessive evaporation of tears, or irregularity of tear component [1]. The symptoms include itching, redness, irritation, ocular fatigue, dryness, and foreign body sensation [1,2]. DED can cause severe pain and limit daily activities such as reading books or using the computer for a long time [3]. These detrimental effects can lead to psychological complications and reduce the quality of life for DED patients [4].

*Achyranthes japonica* is a perennial herb of *Achyranthes* genus [5,6]. *Achyranthis radix*, the roots of *Achyranthes japonica*, has been used in traditional medicine for arthritis, menses, and edema [7]. Recently, the protective effect of *Achyranthis radix* extract (USL) on the eye components has been reported [8]. According to the literature, 1% USL improved the symptoms of dry eye syndrome caused by fine dust [8]. However, the lack of information about the eye drop formulation of USL has limited its clinical application. Thus, the development of eye drop formulations of USL should be conducted prior to the application of USL in DED patients.

Tear deficiency is one of the most common causes of DED. For the treatment of DED, tear substitutes and tear-stimulating methods have been used [9]. In the market, there are various eye drop products in the form of over-the-counter (OTC) drugs, often termed artificial tears. Tear replacement with ocular lubricants and viscosity-enhancing agents such as carboxymethyl cellulose (CMC), hydroxypropyl methylcellulose (HPMC), polyvinyl alcohol (PVA), polyvinylpyrrolidone (PVP), and hyaluronic acid (HA) have been traditionally considered a mainstay of DED therapy. Physicochemical properties of these ocular lubricants have been researched in many studies [10,11,12]. It has been known that the lubricants affect the ocular surface via various mechanisms such as promoting tear retention, protecting the ocular surface, increasing tear film thickness, and relieving dry eye symptoms [13]. The lubricants also increase the precorneal residence time of active ingredient-loaded eye drop formulation and improve bioavailability by enhancing formulation viscosity [14,15].

As described above, various substances such as USL or omega-3 fatty acids have been investigated as potential candidates for the treatment of DED. However, the lack of information about the formulation has been limited to the clinical application of active ingredients for the DED. Thus, formulation optimization using lubricants should be conducted before the clinical application of the active ingredient. The aim of this study was to develop USL-loaded eye drop formulation to show significant cytoprotective and anti-inflammatory effects in conjunctival epithelial cells using various lubricants for DED.

## 2. Materials and Methods

### 2.1. Materials

USL was provided from the Korea Institute of Oriental Medicine (Daejeon, Korea). Acetic acid, monosodium phosphate, disodium phosphate, sodium chloride, hydrochloric acid, benzalkonium chloride, and sodium hydroxide were purchased from Samchun Chemical (Pyungtaek, Korea). Ecdysterone as a reference substance, dimethyl sulfoxide (DMSO), 3-(4,5-dimethylthoazol-2yl)-2,5-diphenyl-2H-tetrazolium bromide (MTT), and sodium CMC (average M_w_ ≒250,000, degree of substitution 0.7) were purchased from Sigma-Aldrich (St. Louis, MO, USA). Dulbecco’s modified Eagle’s medium (DMEM), fetal bovine serum (FBS), penicillin-streptomycin, and trypsin- ethylenediaminetetraacetic acid (EDTA) were purchased from Gibco BRL (Gaithersburg, MD, USA). Metolose 60SH-4000 (HPMC) was provided from Shin-Etsu Chemical Co., Ltd. (Tokyo, Japan). Hyaluronic acid was provided from SK Bioland (Cheonan, Korea). High-performance liquid chromatography (HPLC)-grade acetonitrile (ACN) was obtained from JT Baker (Phillipsburg, NJ, USA). Cyclosporine A (CsA) was purchased from Allergan Co., Ltd. (Dublin, Ireland). All other chemicals were commercial products of analytical or reagent grade and used without further purification.

### 2.2. High-Performance Liquid Chromatography (HPLC)

USL used in the study consisted of various compounds such as ecdysterone, 25R-inokosterone, and 25S-inokosterone. According to the HPLC analysis, ecdysterone was the most abundant compound in USL [16]. Therefore, ecdysterone was used as a marker compound to assay USL. The amount of ecdysterone was determined by reversed-phase HPLC with modification of the previously described method [17]. HPLC analysis of ecdysterone was performed with the Shimadzu LC-2030c 3D (Shimadzu Corporation, Kyoto, Japan) equipped with a UV detector. The analytical column was a C_18_ column (Xterra RP18, 4.6 × 250 mm, 5 μm; Waters, Milford, MA, USA) and the mobile phase consisted of 0.1% acetic acid in distilled water (DW) (mobile phase A) and ACN (mobile phase B). Analysis was conducted by a gradient elution at a flow 1.0 mL/min (B% (time): 10% (0 min)→10% (5.0 min)→20% (6.0 min)→20% (15.0 min)→50% (16.0 min)→50% (25.0 min)→10% (28.0 min)→10% (33.0 min)). The column temperature was maintained at 40 °C and the injection volume was 20 μL. The UV absorbance of ecdysterone was set at 245 nm. All analytic samples were prepared with DW.

### 2.3. Preparation of Achyranthis Radix Extract

USL was obtained from *Achyranthis radix* by hot water extraction. Firstly, 5 kg of *Achyranthis radix* was weighed and added into 40 L of DW, primarily. Then, the mixture was boiled at 100 °C for 3 h and then cooled. The extract was filtered through a membrane filter. The filtrate was lyophilized for 3 days and the obtained powder (about 40% yield) was packed in a sealed state.

### 2.4. Pigmentation Assay with the Egg Shell Membrane

The pigmentation of USL was determined by measuring the color on the membrane. The color measurement was recorded with specular component included value, which was displayed on the spectrophotometer. Briefly, the inner membrane was carefully separated from the cuticula of a boiled egg shell. Then, the separated membrane was immersed in DW until the assay was completed to prevent the membrane from drying. USL solutions were individually prepared with the various concentration of USL and lubricants. The membranes were immersed in each USL solution, taken out at a scheduled time point, washed once with DW, and dried at room temperature (RT). The dried membranes were detected using the spectrophotometer CM-2600d (Konica Minolta, Tokyo, Japan).

In the desorption study, the membrane was first immersed in a 1% USL solution for 15 min, and then the membrane was placed in 100 mL DW. The washing medium was stirred with a magnetic bar with constant stirring speed (300 rpm) at RT. The washed membranes were taken out at the scheduled time-point and measured in the same manner as above. All measurements were repeated three times.

### 2.5. Preparation of Eye Drop Formulations

Four formulations were prepared according to each composition (Table 1). The amount of lubricant was based on common OTC artificial tears products, followed by CMC 0.5%, HA 0.3%, or HPMC 0.3%. When the above amount of lubricant was used, it was confirmed to have the most similar viscosity, thereby minimizing the change in the experimental results due to the difference in viscosity depending on the type of lubricant (Figure S1). The formulations were prepared as a general eye drop manufacturing process with osmotic pressure of about 300 mOsm/L using NaCl. The reported normal tear tonicity was 300–307 mOsm/L in a previous study [18]. Briefly, USL and additives were completely dissolved in DW and then the solution was filtered through a 0.45 μm syringe filter (GE Healthcare, Chicago, IL, USA). Formulations without USL were prepared in the same way. After preparation of eye drop formulations, their physicochemical properties were investigated. The pH, osmolality, and viscosity were measured using pH Meter (827 pH lab, Metrohm, Swiss), Osmomat 3000 (Gonotech GmbH, Berlin, Germany), and rotational viscometer (m-VROC, RheoSense Inc., San Ramon, CA, USA), respectively.

### 2.6. Measurement of Transmittance

Transmittance was measured by calculating the percentage transmittance using microplate reader (Infinite M200 PRO, Tecan Trading AG, Männedorf, Switzerland), with DW as the blank [19]. A total of 100 µL of each sample was placed in a 96-well plate. The transmittance was calculated using the following equation:

$$Transmittance\;\left(\%\right)=\;10^{-\left(OD_{sample}-OD_{blank}\right)}$$

where *OD_sample_* and *OD_blank_* are the absorbance intensity of sample and DW at 600 nm, respectively.

### 2.7. Evaluation of Water Retention

Water retention ability of each formulation was evaluated with a modification of the previously described method [20]. A total of 200 µL of each formulation was dropped onto 2.5 cm diameter filter paper (Millipore filter 3.0 μm Type SS; Merck Millipore, Burlington, NJ, USA) on the weighing dish. Each formulation-soaked filter paper and weighing dish were immediately weighed, which was determined as the value at time point 0. The weight of each sample was measured at 0, 5, 10, and 20 min. The experiments were conducted at RT (25 °C) in 47% of relative humidity (RH).

### 2.8. Conjunctival Epithelial Cell Studies

The conjunctival epithelial cells were purchased from the American Type Culture Collection (ATCC) (Manassas, VA, USA). Cells were maintained in ATCC Mammary Epithelial Cell Basal Medium (Manassas, VA, USA) containing ATCC Mammary Epithelial Cell Growth Kit (Manassas, VA, USA).

#### 2.8.1. Cytotoxicity Study of Formulations

The cytotoxicity of formulations with/without USL against conjunctival epithelial cells were determined using MTT assay. Conjunctival epithelial cells were cultured in DMEM at 37 °C in a 5% CO_2_ incubator. The medium was supplemented with 10% (*v*/*v*) FBS and 100 units/mL of penicillin-streptomycin. The cells were seeded into 96-well plates at a density of 3 × 10^4^ cells/well in 100 μL DMEM and incubated at 37 °C in a 5% CO_2_ incubator overnight to promote adhesion.

Samples were prepared as follows. Firstly, the composition of the formulations was dissolved in DMEM instead of DW. Then, samples diluted with DMEM were incubated with the cells for 6 h. After incubation, 30 μL of MTT solution (5 mg/mL) was added to each well and the cells were incubated. After 2 h, the medium was removed. Two hundred microliters of DMSO was added into each well to dissolve formazan crystals. Finally, the absorbance of each well was measured at 560 nm using a microplate reader. The cell viability was calculated by using the following equation:

$$\mathrm{Cell}\;\mathrm{viability}\;\left(\%\right)=100\times \frac{OD_{sample}}{OD_{control}}$$

where *OD_sample_* is the absorbance intensity of the wells treated with samples, and *OD_control_* is the absorbance intensity of the wells incubated with the DMEM without USL.

#### 2.8.2. Evaluation of Protective Effect on Conjunctival Epithelial Cells against Dehydration

Protective ability of each formulation against dehydration of conjunctival epithelial cells was evaluated with modification of a previously described method [20]. The cells were seeded into 96-well plates at a density of 3 × 10^4^ cells/well in 100 μL DMEM and incubated at 37 °C in a 5% CO_2_ incubator overnight to promote adhesion. During cell culture, media was removed and the cells were pre-incubated with dilutions of the formulations (samples) for 30 min in triplicate. The samples were removed and the cells were exposed to air for 5, 15, 30, and 60 min. Then, the cells were incubated with DMEM for 1 h. After incubation, 30 μL of MTT solution (5 mg/mL) was added to each well and the cells were incubated for 2 h. The subsequent procedure was the same as described above.

#### 2.8.3. Effect of Formulations on Hyperosmotic Stress-Stimulated Human Conjunctival Epithelial Cells

Inhibitory effect of formulations on hyperosmotic stress-induced inflammation was assessed with modification of a previously described method [21]. The cells were co-treated with the indicated concentration of formulations and hyperosmolar cell culture media (528 mOsM) for 24 h. Total RNA was extracted using TRIZOL, and the yield and purity of RNA were conducted by the measurement of ratio of the absorbance at 260 and 280 nm. The quantitated RNA was synthesized using a SuperScript II kit for complementary DNA (cDNA). The cDNA was examined by quantitative real-time polymerase chain reaction (PCR) using specific primers listed in Table 2 by thermocyclers from Bio-Rad.

### 2.9. Stability Studies

#### 2.9.1. Long-Term and Accelerated Test

The long-term and accelerated test was carried out by storing formulation in 25 ± 2 °C/60% ± 5% RH and 40 ± 2 °C/60% ± 5% RH for 4 weeks to measure the several parameters including content change of ecdysterone and formation of the precipitate. The sterility test was conducted using fluid thioglycolate medium and soybean-casein digest medium as reported in the Korea Pharmacopoeia to confirm the absence of the aerobic and anaerobic bacteria and fungi. In brief, thioglycolate medium and soybean-casein digest medium were dissolved in DW and then autoclaved. A total of 1 mL of 1% USL was transferred into 12 mL of media. Then, the media were incubated at 32 °C and the growth of micro-organisms was observed for 14 days.

#### 2.9.2. Compatibility Test

The compatibility test between USL and additives was conducted as the same method for accelerated test and evaluated for the aspect of content and transmittance. In the compatibility test, 1% USL and each additive were stored together at 40 °C oven for 4 weeks.

#### 2.9.3. Thermal Stability Test

The stability of the formulation against heat was evaluated. Each formulation was assessed for transmittance by measuring the amount of precipitate formed at 121 °C for 30 min. Transmittance was measured as described above.

### 2.10. Statistical Analysis

Data were expressed as mean ± SD. Statistical analysis was conducted using GraphPad Prism 5.0 software (San Diego, CA, USA). Student’s *t*-test was used to compare two different groups of samples. Differences among multiple groups were performed by one-way ANOVA followed by Tukey’s multiple comparisons. * *p*-value < 0.05, ** *p*-value < 0.01, and *** *p*-value < 0.001 were considered significant.

## 3. Results and Discussion

### 3.1. Quantification of Ecdysterone in USL

Figure S2 shows HPLC chromatograms of the ecdysterone marker compound, USL solution, and F1. Ecdysterone exhibited a retention time of 12.8 min (Figure S2A). Moreover, this peak was also confirmed in the USL solution and USL eye drop formulation, F1 (Figure S2B,C). In this study, ecdysterone was used as a marker compound for USL and 1% USL solution contained 14.37 ± 0.04 μg/mL of ecdysterone on average. On the basis of this, the following studies confirmed the USL content through the concentration of ecdysterone.

### 3.2. Physicochemical Properties of USL Eye Drop Formulations

The developed formulations were characterized for various physicochemical properties including clarity, pH value, viscosity, and osmolarity (Table 3). All the formulations were clear. When the pH and osmolality of the USL eye drop formulations differ significantly from tears, it may cause eye damage or discomfort [22]. The pH value and osmolarity of formulations were varied from 6.71 to 6.75 and 307 to 315 mOsm/kg. Regardless of the type of lubricants, the formulations similarly showed moderate viscosity (2-4 cP), indicating that the formulation was free from immediate drainage or blinking pain due to abnormal viscosity.

### 3.3. Pigmentation Assay with Egg Shell Membrane

Because the USL solution had a yellowish color, there was a possibility of pigmentation in the cornea when it was used as eye drops. A new method was developed to identify the possibility of pigmentation with a little modification [21,23]. The egg shell inner membrane could be used as a protein membrane to simply confirm the pigmentation. In previous studies, the physical properties of the egg shell membrane and its potential as a dye adsorbent have been studied [23,24]. The egg shell membrane is an amorphous natural biomaterial with a porous and fibril structure with adsorption properties. It also has a complex lattice of stable and insoluble fibers [25,26]. Despite its various advantages, there has been no study evaluating the chromaticity of the adsorbed material. The egg shell was used for the reason that it is easily obtained and because it is easy to identify the pigmentation because of its white color. The pigmentation was evaluated by measuring the color on the membrane using a spectrophotometer. Spectral reflectance values from the basic colorimetric quantities can be calculated by the International Commission on Illumination (CIE) system and transformed into CIE L*, a*, and b* [27,28,29]. L* (brightness) can range from “0” black to “100” white and constitutes a vertical axis in 3-dimensional color space. The a* and b* constitute horizontal axes, and represent green-red (-a* to +a*) and blue-yellow (-b* to +b*), respectively [30]. The egg shell inner membrane, not pigmented, showed L*, a*, and b* values of 94.3 ± 0.3, −0.7 ± 0.1, and −0.8 ± 0.4, respectively, indicating that the egg shell membrane had a bright white color. In this study, the value of a* was not changed significantly, so only L* and b* were considered.

**Figure 1:**
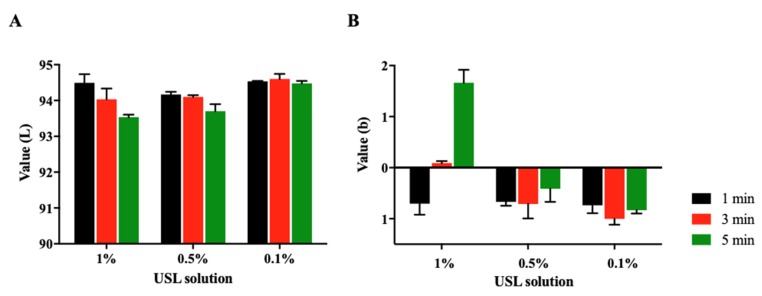
Pigmentation evaluation by measuring the color change on the egg shell membrane according to USL concentration (0.1%, 0.5%, and 1%) and incubation time (1, 3, and 5 min). (**A**) L* value, (**B**) b* value (*n* = 3).

Because this method was newly developed, the sufficient validation and verification of the method was necessary. Firstly, the pigmentation was evaluated by measuring the color change on the egg shell inner membrane according to the concentration of USL and incubation time (Figure 1). In the 1% USL group, pigmentation occurred with increased incubation time; L* was decreased and b* was increased. It was found that 1% USL with 5 min incubation led the egg shell inner membrane to become brighter and yellowish. On the other hand, 0.5% and 0.1% USL solution did not create pigmentation; L* and b* were not changed significantly up to 5 min. It was suggested that less than 1% USL did not affect the pigmentation, even if the contact time into egg shell inner membrane was increased up to 5 min.

**Figure 2:**
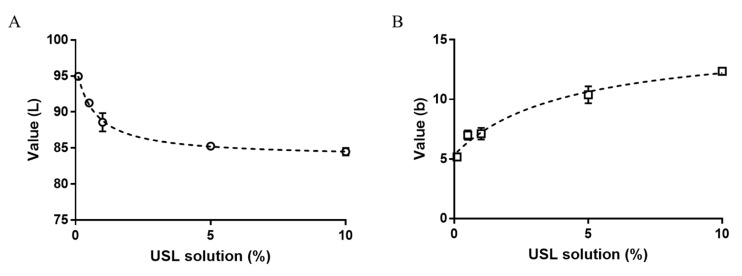
Pigmentation evaluation by measuring the color change on the egg shell membrane according to USL solution concentration (%): (**A**) L* value, (**B**) b* value. The R^2^ values were 0.9778 and 0.9631 for A and B, respectively (*n* = 3).

In addition, the change of L* value and b* value according to the amount of lubricants and USL concentration was confirmed in the state of increasing the adsorption time to 30 min to verify the analysis assay. Using 1% of USL solution, the presence of lubricants clearly induced more pigmentation at the same time, which resulted in a greater change in L* and b* values (Figure S3A,B). Nevertheless, depending on the amount of lubricants, there were no differences in the values due to the pigmentation. This was because the viscosity that can be used as an eye drop is limited to about 20 cP and the change therein cannot have a great influence on the pigmentation. Meanwhile, the pigmentation evaluation results with various USL solution concentrations of 0.1%, 0.5%, 1%, 5%, and 10% showed a clear tendency (Figure 2). Even at the same time of 30 min, the decrease of L* value and the increase of b* value tended to be higher when the concentration of USL was high. However, the change of L* and b* values was not linearly proportional to the increase in concentration, because when the concentration was above a certain level, the L* and b* values did not change very much. This was because the adsorption capacity of the egg membrane used in the experiment was limited. As a result, the graph showed a non-linear tendency, and the R^2^ values of L* and b* values were 0.9778 and 0.9631, respectively.

**Figure 3:**
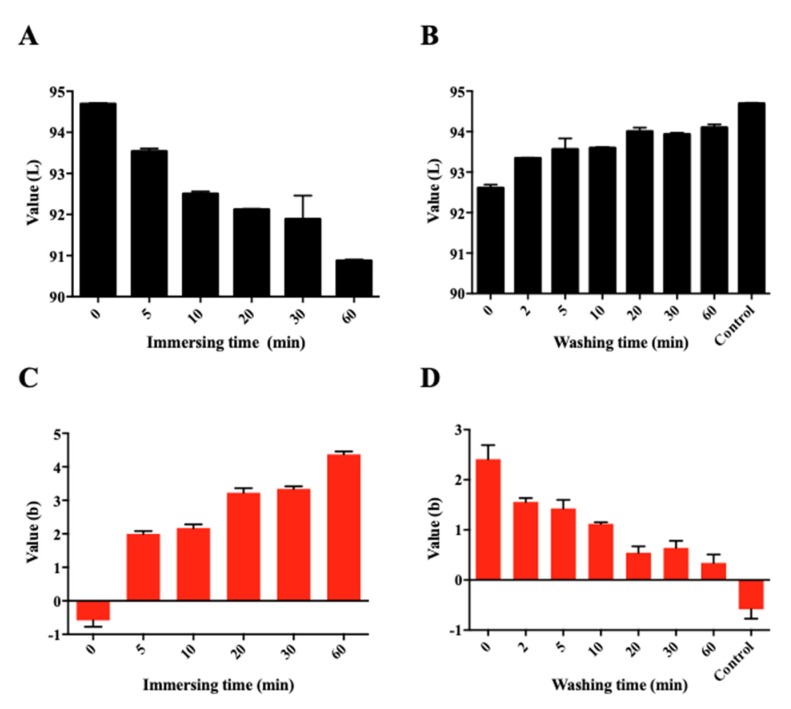
Pigmentation evaluation by measuring the color change on the egg shell inner membrane according to time in 1% USL solution (**A**,**C**) and according to washing time after immersing the membrane in 1% USL solution for 15 min (**B**,**D**) (*n* = 3). (**A**,**B**) L* value, (**C**,**D**) b* value.

When the concentration of USL was fixed to 1% and contact time was increased, pigmentation was observed to be constantly increased over a long time (Figure 3A,C). The desorption test was conducted to determine whether pigmentation occurred irreversibly and to mimic the environment in vivo (real eye). Figure 3B,D shows that the pigmented color was faded during the wash time; the L* value increased (increased whiteness) and b* value decreased (decreased yellowness) again. This meant that pigmentation is a reversible phenomenon and may be transient. It was assumed that the pigmentation was caused by embedding in protein structures and could be released simply. It is expected that severe pigmentation will not occur in the clinical usage condition of USL.

This assay is a new method for assessing the pigmentation of drugs for eye drop development in vitro. However, unlike the cornea, the egg shell membrane is a denatured protein, and this assay has not been proven to be correlated with in vivo study. Therefore, in vivo studies using animal models should be followed and correlation with in vitro and in vivo studies should be confirmed in further research. Nevertheless, this study proved that this novel assay is logically reasonable enough through sufficient experiment and validation. As a result, pigmentation assay with egg shell membrane can be used as one of the candidates for conducting experiments on adsorption and desorption of pigments loaded in the eye drop formulation in situations where animal testing is limited.

### 3.4. Evaluation of Water Retention Ability

**Figure 4:**
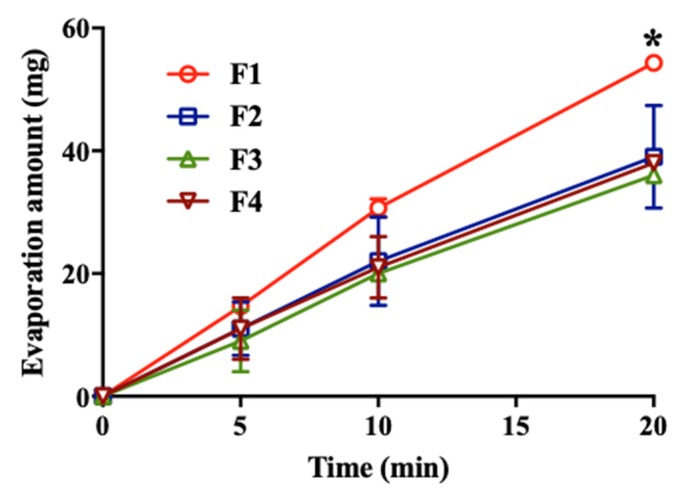
Water retention ability of formulations. The filter papers were soaked with 0.2 mL of each of the lubricants and left in an open container at 25 °C and a humidity of 47%. * *p* < 0.05 versus F1 to the other formulations.

The water retention ability of each formulation was evaluated because residual water in the eye critically affects the severity of DED. The evaporation of water from the membrane proceeded over time, and the interaction between the membrane and the formulation influenced evaporation. It was observed that the evaporation amount increased linearly until 20 min. F2, F3, and F4 containing each lubricant showed inhibition of moisture evaporation compared to F1 (Figure 4). At 20 min, the amount of evaporation was significantly different from that of the other formulations correlated with a previous study [20]. This could be explained by the viscosity, which contributed to enhancing the drug residence time [31]. The water retention ability of lubricants can increase the absorption of active ingredients and have effectiveness in protecting dryness [17].

### 3.5. Conjunctival Epithelial Cell Studies

**Figure 5:**
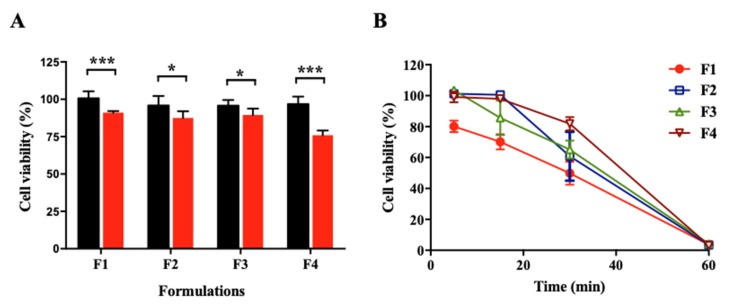
Cytotoxicity of formulations and formulations without USL in conjunctival epithelial cells for 6 h (*n* = 3). (**A**) Protective effect of formulations from dehydration according to time. Cells were pre-incubated with the formulations for 30 min and placed under dehydrating conditions (*n* = 3). (**B**) Black or red bars represent the formulation with or without USL, respectively. * *p* < 0.05, significantly different from the formulation with USL. *** *p* < 0.001, significantly different from the formulation with USL.

The cytotoxicity of the formulations was evaluated with conjunctival epithelial cells (Figure 5A). The formulations did not show cytotoxicity when incubated for 6 h, but the formulations without USL were significantly cytotoxic (cell viability of F1, F2, F3, and F4 without USL was 91.1% ± 1.1%, 87.6% ± 4.5%, 89.5% ± 4.3%, and 76% ± 3.2%, respectively). According to Doktorovova et al. (2014) [32], in cytotoxicity assay, when the cell viability of materials is greater than 70%, the materials are generally considered as non-toxic to cells. In this study, cellular viability was greater than 75% for all formulations. However, the cell viability of formulation without USL was significantly lower than that of formulation with USL. This phenomenon might have been due to the protective effect of USL on conjunctival epithelial cells. It has been reported that the apoptotic death of corneal cells reduced after the administration of USL [16]. In addition, an anti-apoptotic effect of ecdysterone has been reported [33]. Thus, USL used in this study, which consisted of ecdysterone, seems to improve cell viability. In the cytotoxicity study, experiments were carried out with various concentrations of formulations (data not shown). Because the three-fold diluted solution of formulations was the highest concentration that did not show cytotoxicity, cell dehydration study was conducted with the three-fold diluted solution of formulations.

The protective effect against dehydration was evaluated by exposing conjunctival epithelial cells to dry air (Figure 5B). Cells were pre-incubated with the formulations and dried for a scheduled time. Cells had a reduced survival rate over time according to drying time and did not survive in the drying condition for 60 min. Specifically, the cells were no longer viable when the medium was dried out. F2, F3, and F4, containing each lubricant, had a significantly higher protective effect compared with F1 after 5, 15, and 30 min of dehydration (*p* < 0.05 at 5 min, F1 vs. F2, F3, and F4; at 15 min, F1 vs. F2 and F4; at 30 min, F1 vs. F3 and F4). No significant difference was observed between F2, F3, and F4 at all time points. In other words, formulations containing lubricant and acting water retention agent can reduce cell damage in DED. In particular, F4 tended to more effectively protect cells from dehydration until 30 min compared with the others. These results exhibited a correlation with the result of the water retention ability test.

### 3.6. Effect of Formulations on Hyperosmotic Stress-Stimulated Human Conjunctival Epithelial Cells

**Figure 6:**
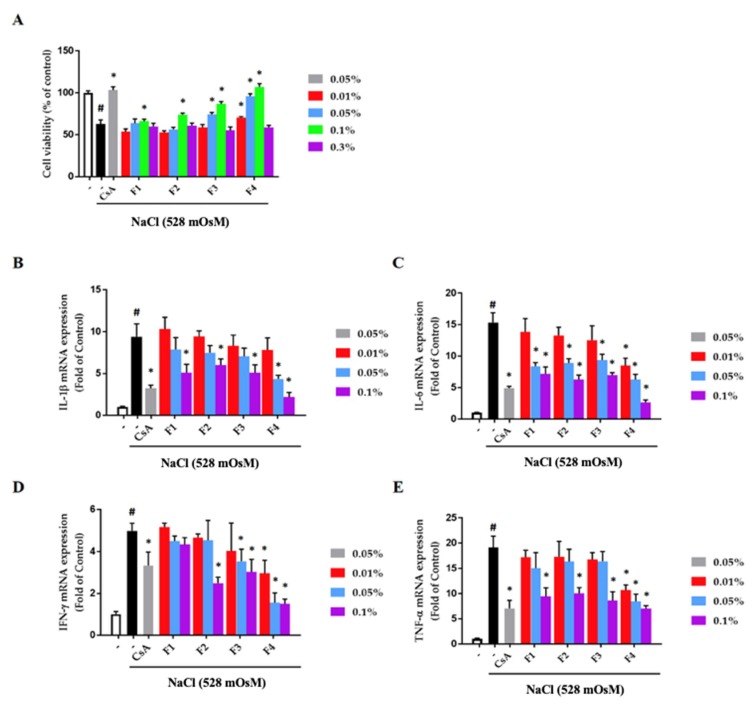
Effect of formulations on hyperosmolar stress-stimulated inflammation in human conjunctival epithelial cells. (**A**) The cells were co-treated with each formulation and hyperosmolar cell media (528 mOsM) for 24 h (*n* = 4). mRNA expression of IL-1β (**B**), IL-6 (**C**), IFN-γ (**D**), and TNF-α (**E**) was examined by quantitative real-time PCR assay. Cyclosporine A (CsA) was used as a positive control. Glyceraldehyde 3-phosphate dehydrogenase (GAPDH) was regarded as an internal control (*n* = 3). # *p* < 0.05, significantly different from the untreated group; * *p* < 0.05, significantly different from the hyperosmotic-treated group.

Hyperosmotic stimuli induce the severe damage to the conjunctival epithelium through the production of inflammatory cytokines [34]. To examine the effect of formulations on the hyperosmotic stress-induced cytotoxicity in human conjunctival epithelial cells, cell viability assay was performed at the indicated concentrations with sodium chloride-containing cell culture media (528 mOsM) for 24 h. As shown in Figure 6A, hyperosmolar cell culture media significantly ameliorated the cell viability (62.9% ± 4.9%, *p* < 0.001), but cell viability was remarkably restored by each formulation at a concentration of 0.1%. Moreover, F4 had a critical recovery effect of cell viability from low concentrations. However, there was no effect of the recovery of cell viability in the formulation at 0.3% concentration. To determine whether formulations attenuated on inflammation in hyperosmotic stress-induced human conjunctival epithelial cells, quantitative real-time PCR was conducted. The mRNA of each of the inflammatory cytokines (interleukins-1β (IL-1β), IL-6, interferon-γ (IFN-γ), and tumor necrosis factor-α (TNF-α)) was significantly increased by hyperosmolar cell culture media. In the highest concentration of formulations, each inflammatory cytokine was considerably inhibited. Also, treatment of F4 exhibited a significant inhibitory effect on inflammation with a concentration-dependent manner compared with the other formations (Figure 6B–E). In particular, F4 was found to have a significant anti-inflammatory effect in the hyperosmotic stress-stimulated human conjunctival epithelial cells compared with cyclosporine A (CsA), a positive control, which is well known for the treatment of dry eye disease.

### 3.7. Stability Studies

#### 3.7.1. Long-Term and Accelerated Test

In order to ensure the quality, safety, and efficacy during the shelf life, long-term and accelerated tests were performed for 4 weeks at 25 ± 2 °C/60% ± 5% RH and 40 ± 2 °C/75% ± 5% RH each (Table 4). There was no change in every formulation in terms of color, odor, and homogeneity. However, the pH value slightly decreased and osmolality slightly increased. It was confirmed that there was no difference between the formulations, but the change was more significant at 40 °C than 25 °C. Although it should be noted that even with the decrease in pH and increase in osmolality, the data were reasonably within the official acceptable range in order to avoid the eye damage or discomfort. In addition, the transmittance and the net content were checked to confirm the precipitation and the decrease of the content of the USL during the storage period. As a result, both transmittance and content were changed to less than ±2%, and this difference was found to be insignificant. In addition, the sterility test over 24 h confirmed that the sterile state of the formulation was maintained even in long-term or accelerated conditions. This result indicates that the USL eye drop formulation was sufficiently safe during the storage period.

#### 3.7.2. Compatibility Test

Because various components such as saccharides, saponins, phytosterols, phytoecdysteroids, and proteins were contained in USL, studies on solution stability and compatibility with additives were carried out [35,36]. All additives used in the formulations did not cause any change in the content of the ecdysterone or formation of the precipitate when the compatibility test was conducted in a 40 °C oven for 4 weeks. Benzalkonium chloride, a cationic surfactant, is a commonly used preservative in eye drop products [10]. Therefore, the development of formulations containing benzalkonium chloride was attempted. However, when 0.01% benzalkonium chloride and 1% USL were stored in solution, a large amount of precipitate was formed (transmittance; 82.2% ± 1.6%) (Table S1). It was assumed that the precipitate was formed because of the interaction between the protein in USL and benzalkonium chloride used as a protein precipitation agent [37]. Also, benzalkonium chloride had high cytotoxicity to conjunctival epithelial cells [38,39]. When conjunctival epithelial cells were incubated with 0.01%, 0.0033%, 0.0011%, 0.00037%, 0.00012%, and 0.000041% benzalkonium chloride for 12 h, cell viability was 7.7% ± 0.1%, 7.3% ± 0.4%, 10.3% ± 3.9%, 41.0% ± 7.4%, 79.0% ± 8.3%, and 93.8% ± 4.4%, respectively, and thus benzalkonium chloride was not considered as an additive in this study (Figure S4).

#### 3.7.3. Thermal Stability Test

**Figure 7:**
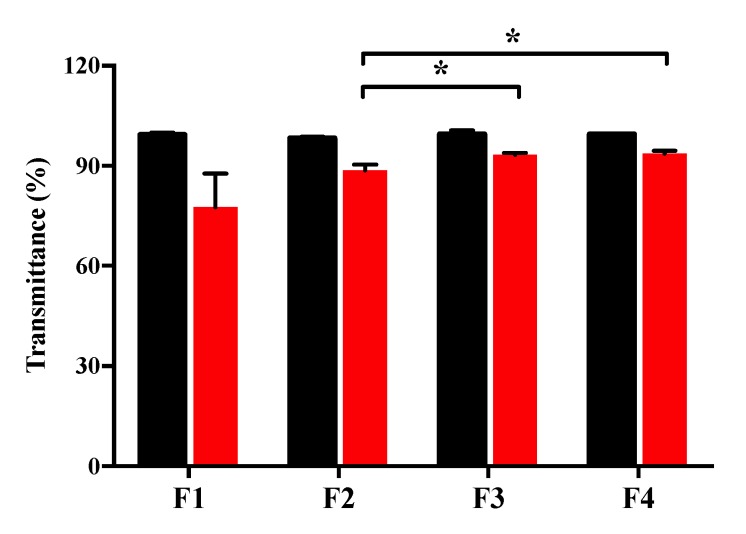
Stability evaluation of precipitation after thermal condition (121 °C, 30 min) of each formulation (*n* = 3). Black or red bars represent the pre- or post-heating condition, respectively. * *p* < 0.05, significantly different from the F2 formulation.

Figure 7 shows that the thermal stability of formulations in the sterilization conditions (121 °C, 30 min). After the thermal process, the transmittance of F1, F2, F3, and F4 was changed to 77.7% ± 10.1%, 88.6% ± 1.8%, 93.3% ± 0.6%, and 93.6% ± 0.9%, respectively. In the preliminary experiments, no precipitate was formed after this thermal process in the formulations without USL, and thus the formation of precipitates in F1, F2, F3, and F4 was probably due to the denaturation of the protein in USL (Figure S5). However, the precipitation was decreased when formulations contained each lubricant. In particular, among the formulations containing lubricants, F3 and F4 exhibited better precipitation-inhibiting effects than F2. It was reported that the stability of protein in thermal conditions increases when polymers or sugars are added [40,41,42,43,44]. This thermal resistance can maintain the quality of the eye drop when the formulation is exposed to unexpected thermal conditions.

## 4. Conclusions

In this study, USL-loaded eye drop formulations were developed using different lubricants. When the formulations with or without USL were compared, USL showed significant cytoprotective and anti-inflammatory effects in conjunctival epithelial cells. The lubricants used in this study effectively inhibited water evaporation in the water retention study and allowed cells to survive longer from drying condition in the cell dehydration study. In particular, F4 exhibited a significant inhibitory effect on inflammation in a concentration-dependent manner compared with other formations. In future studies, this formulation should be evaluated to prove a potential in vivo efficacy of the formulation in DED.

## Figures and Tables

**Table 1:** Composition of each formulation.

Compound	F1	F2	F3	F4
USL	100 mg	100 mg	100 mg	100 mg
CMC		50 mg		
HA			30 mg	
HPMC				30 mg
Monosodium phosphate	50 mg	50 mg	50 mg	50 mg
Disodium phosphate	50 mg	50 mg	50 mg	50 mg
NaCl	40 mg	40 mg	40 mg	40 mg
DW (q.s.)	10 mL	10 mL	10 mL	10 mL

*Achyranthis radix* extract (USL); carboxymethyl cellulose (CMC); hydroxypropyl methylcellulose (HPMC); hyaluronic acid (HA); distilled water (DW); quantum sufficit (q.s.).

**Table 2:** Real-time PCR primer sequences.

Genes	Sequence (5′→3′)	
IL-1β	Sense	5′- ACAGATGAAGTGCTCCTTCCA-3′
	antisense	5′-GTCGGAGATTCGTAGCTGGAT-3′
IL-6	Sense	5′-AAATTCGGTACATCCTCGAC-3′
	antisense	5′-CAGGAACTGGATCAGGACTT-3′
IFN-γ	Sense	5′-TCCCATGGGTTGTGTGTTTA-3′
	antisense	5′-AAGCACCAGGCATGAAATCT-3′
TNF-α	Sense	5′-TTCTCCTTCCTGCTTGTG-3′
	antisense	5′-CTGAGTGTGAGTGTCTGG-3′
GAPDH	Sense	5′-CCAGCCGAGCCACATCGCTC-3′
	antisense	5′-ATGAGCCCCAGCCTTCTCCAT-3′

**Table 3:** Physicochemical properties of *Achyranthis radix* extract (USL) eye drop formulations.

Formulations	F1	F2	F3	F4
Clarity	Clear	Clear	Clear	Clear
pH value	6.71 ± 0.02	6.74 ± 0.02	6.75 ± 0.01	6.75 ± 0.00
Osmolality (mOsm/kg)	307 ± 0.82	315 ± 2.49	309 ± 5.44	313 ± 4.50
Viscosity (cP)	-	2.183	3.663	3.267

**Table 4:** Long-term and accelerated test of USL eye drop formulations for 4 weeks.

Parameters	Storage Condition							
	25 ± 2 °C/60% ± 5% RH				40 ± 2 °C/75% ± 5% RH			
	F1	F2	F3	F4	F1	F2	F3	F4
Color	No change in color				No change in color			
Odor	No change in odor				No change in odor			
Homogeneity	Smooth				Smooth			
pH	6.44 ± 0.01	6.40 ± 0.01	6.45 ± 0.02	6.38 ± 0.01	5.89 ± 0.01	5.70 ± 0.01	6.01 ± 0.01	5.87 ± 0.01
Osmolarity (%)	330 ± 3	319 ± 8	344 ± 4	323 ± 11	360 ± 9	379 ± 12	381 ± 7	365 ± 14
Transmittance	99.47 ± 0.12	99.07 ± 0.24	98.97 ± 0.19	99.93 ± 0.09	99.53 ± 0.07	98.70 ± 0.20	99.10 ± 0.24	99.30 ± 0.18
Net content (%)	98.97 ± 0.30	98.57 ± 0.28	98.73 ± 0.19	99.03 ± 0.09	99.63 ± 033	100.30 ± 0.29	99.53 ± 0.19	98.90 ± 0.17
Sterility test	No microbial growth was observed at 24 h				No microbial growth was observed at 24 h			

## Supplementary Materials

The following are available online at https://www.mdpi.com/1999-4923/12/2/165/s1, **Figure S1.** Effect of amount and type of lubricants on viscosity, **Figure S2.** HPLC chromatograms of (A) ecdysterone marker compound, (B) USL 1% solution, and (C) F1, **Figure S3.** Pigmentation evaluation by measuring the color change on the egg shell membrane according to lubricants amount (A) L* value, (B) b* value, **Figure S4.** Cytotoxicity of USL, F1 and F1 + BZK in conjunctival epithelial cell for 12 h, **Figure S5.** Stability evaluation of precipitation after thermal condition (121 °C, 30 min) of each formulation without USL (n = 3).
