# Supplementary material for: Achyranthis radix Extract-Loaded Eye Drop Formulation Development and Novel Evaluation Method for Dry Eye Treatment
Source: Pharmaceutics. 2020 Feb 17;12(2):165. doi: 10.3390/pharmaceutics12020165 (PMC7076473; doi:10.3390/pharmaceutics12020165)
Supplement: Supplementary file 1 [file pharmaceutics-12-00165-s001.pdf]

Supplemental Results

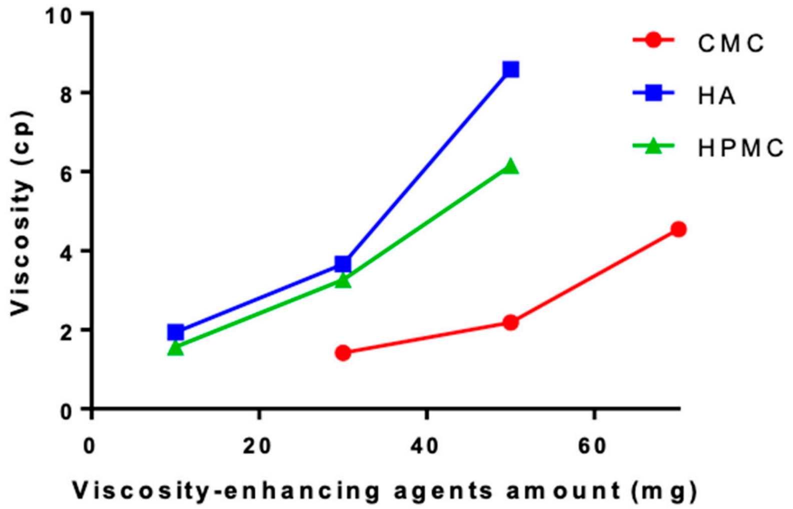

Figure S1. Effect of amount and type of lubricants on viscosity.

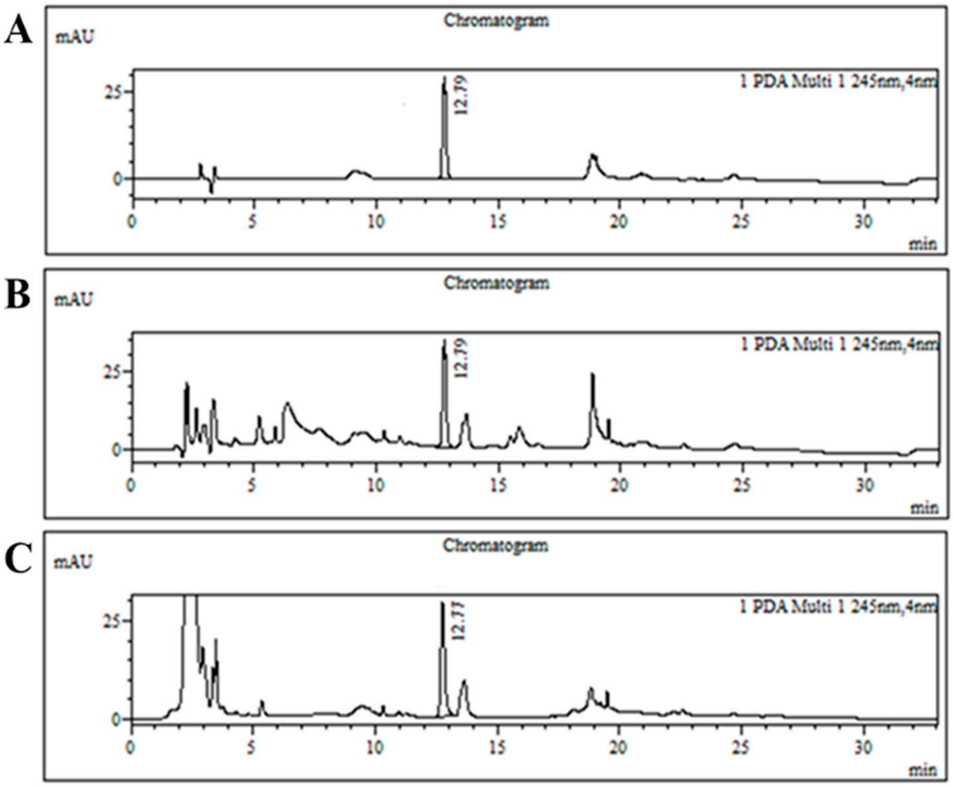

Figure S2. HPLC chromatograms of (A) ecdysterone marker compound, (B) USL 1% solution, and (C) F1.

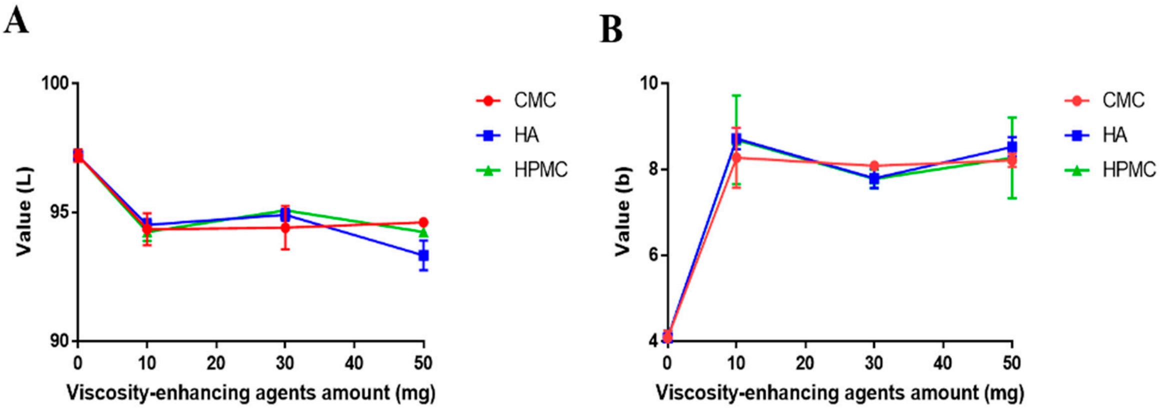

Figure S3. Pigmentation evaluation by measuring the color change on the egg shell membrane according to lubricants amount (A) L* value, (B) b* value.

Table S1. The compatibility with additives data and stability evaluation of formulations in 40°C oven storage for 3 weeks

	Ecdysterone content	Transmittance (600 nm)
**1% USL and 0.3% HA**	100.3 ± 0.4%	98.8 ± 0.1%
**1% USL and 0.3% HPMC**	99.8 ± 0.2%	98.8 ± 0.5%
**1% USL and 0.5% CMC**	99.9 ± 0.8%	98.8 ± 0.3%
**1% USL and 0.9% NaCl**	99.1 ± 0.3%	98.6 ± 0.2%
**1% USL and 0.5% phosphate buffer**	99.8 ± 0.6%	99.2 ± 0.3%
**1% USL and 0.01% BZK**	99.3 ± 0.2%	82.2 ± 1.6%
**F1**	99.6 ± 1.3%	99.5 ± 0.2%
**F2**	100.3 ± 0.5%	98.7 ± 0.2%
**F3**	99.5 ± 1.0%	99.1 ± 0.2%
**F4**	99.1 ± 0.2%	99.3 ± 0.2%

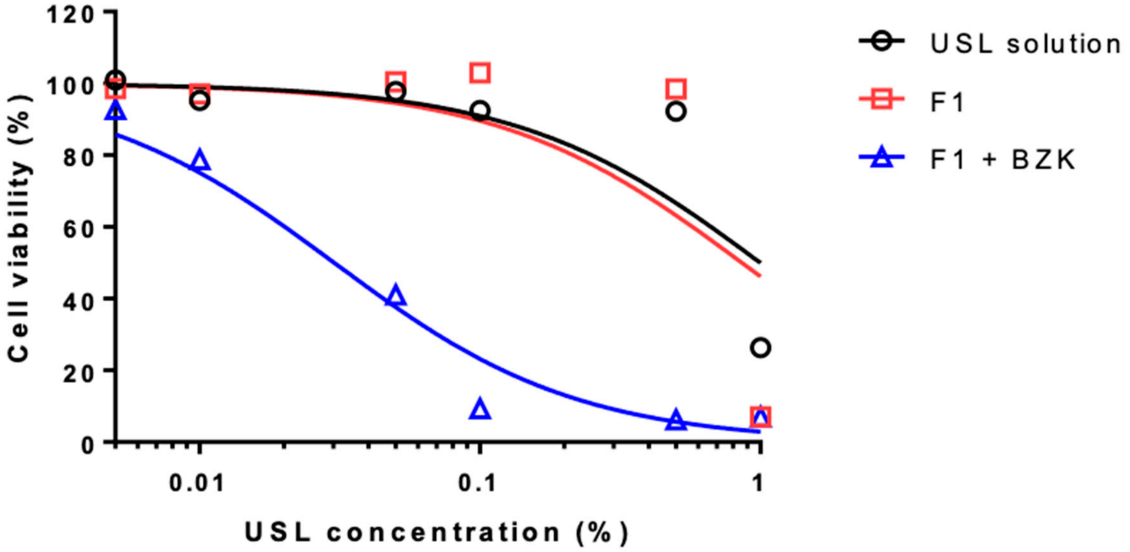

Figure S4. Cytotoxicity of USL, F1 and F1 + BZK in conjunctival epithelial cell for 12 h.

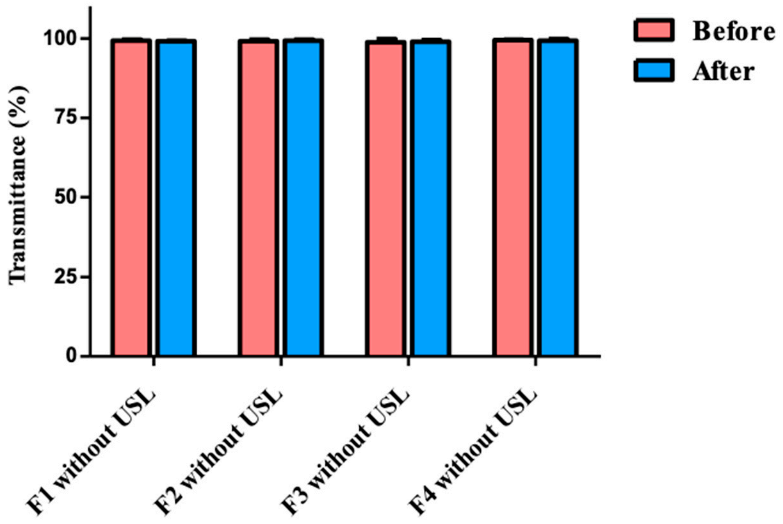

Figure S5. Stability evaluation of precipitation after thermal condition (121°C, 30 min) of each formulation without USL (n=3).
